# Multiple vulnerabilities and maternal healthcare in Vietnam: findings from the Multiple Indicator Cluster Surveys, 2000, 2006, and 2011

**DOI:** 10.3402/gha.v9.29386

**Published:** 2016-02-29

**Authors:** Hoang Van Minh, Juhwan Oh, Kim Bao Giang, Vu Duy Kien, You-Seon Nam, Chul Ou Lee, Tran Thi Giang Huong, Luu Ngoc Hoat

**Affiliations:** 1 Hanoi School of Public Health, Hanoi, Vietnam; 2 JW LEE Center for Global Medicine, Seoul National University College of Medicine, Seoul, Korea; 3 Department of Health Education, Center for Health System Research, Hanoi Medical University, Hanoi, Vietnam; 4 Center for Population Health Sciences, Hanoi School of Public Health, Hanoi, Vietnam; 5 Department of Family Medicine, Seoul National University Hospital, Seoul, Korea; 6 Department of International Cooperation, Ministry of Health, Hanoi, Vietnam; 7 Department of Biostatistics and Health Informatics, Hanoi Medical University, Hanoi, Vietnam

**Keywords:** healthcare, skilled antenatal care, skilled delivery, multiple socioeconomic vulnerabilities, inequity, inequality

## Abstract

**Background:**

Knowledge of the aggregate effects of multiple socioeconomic vulnerabilities is important for shedding light on the determinants of growing health inequalities and inequities in maternal healthcare.

**Objective:**

This paper describes patterns of inequity in maternal healthcare utilization and analyzes associations between inequity and multiple socioeconomic vulnerabilities among women in Vietnam.

**Design:**

This is a repeated cross-sectional study using data from the Vietnam Multiple Indicator Cluster Surveys 2000, 2006, and 2011. Two maternal healthcare indicators were selected: (1) skilled antenatal care and (2) skilled delivery care. Four types of socioeconomic vulnerabilities – low education, ethnic minority, poverty, and rural location – were assessed both as separate explanatory variables and as composite indicators (combinations of three and four vulnerabilities). Pairwise comparisons and adjusted odds ratios were used to assess socioeconomic inequities in maternal healthcare.

**Results:**

In all three surveys, there were increases across the survey years in both the proportions of women who received antenatal care by skilled staff (68.6% in 2000, 90.8% in 2006, and 93.7% in 2011) and the proportions of women who gave birth with assistance from skilled staff (69.9% in 2000, 87.7% in 2006, and 92.9% in 2011). The receipt of antenatal care by skilled staff and birth assistance from skilled health personnel were less common among vulnerable women, especially those with multiple vulnerabilities.

**Conclusions:**

Even though Vietnam has improved its coverage of maternal healthcare on average, policies should target maternal healthcare utilization among women with multiple socioeconomic vulnerabilities. Both multisectoral social policies and health policies are needed to tackle multiple vulnerabilities more effectively by identifying those who are poor, less educated, live in rural areas, and belong to ethnic minority groups.

## Introduction

Vietnam has made significant progress in achieving the Millennium Development Goals (MDGs). Maternal mortality in Vietnam has declined considerably over the last two decades, from 233 per 100,000 live births in 1990 to 69 per 100,000 live births in 2009. Good progress has also been made through increased access to quality reproductive healthcare and improved services for the poor and other vulnerable groups (1). Maternal and neonatal health has received considerable attention by the Vietnamese health system, as reflected in recent policies. For example, Vietnam's Five-Year Socio-Economic Development Plan 2006–2010 clearly specified that improvement in the ‘material and spiritual life’ of women was one of Vietnam's most important priorities (2). The Vietnam National Strategy for Reproductive Health Care 2001–2010 highlighted the nation's overall directions for reproductive health, with improved strategies focused on narrowing disparities between different regions and target groups (3). The National Plan for Safe Motherhood 2003–2010 was also committed to promoting maternal and neonatal health by delivering new tools and equipment to provincial and district-level facilities. The plan also contributed to the following: the expansion of training for doctors and nurses in essential newborn care; the provision of adequate supplies of essential drugs; the education of healthcare professionals; the identification and treatment of anemia; and the prevention of mother-to-child transmission of HIV (4). Vietnam has also implemented a population and reproductive health strategy for 2011–2020, targeting universal coverage for reproductive health (5). In 2012, the Ministry of Health (MOH) issued the Master Plan for Universal Health Coverage from 2012–2015 and 2020 (6–8).

Several studies in Vietnam have reported that inequities exist in access to maternal healthcare between different segments of the population. Low education, poverty, and ethnicity have been shown to be significantly associated with lower antenatal care coverage and skilled birth attendance (9–13). However, little attention has been paid to the assessment of associations between multiple dimensions of (socioeconomic) vulnerabilities and inequities in health. *Vulnerability*, in this context, means susceptibility to health problems, harm, or neglect (14, 15). The concept of *multiple vulnerabilities* has received recent attention by both researchers and policy makers (16–18) because the use of individual-level socioeconomic indicators alone may fail to capture the health impacts of contextual factors (18). Approaches covering multiple vulnerabilities can take into account the effects of the individual as well as the household and contextual disadvantages that impact on health (19, 20).

Equity in health is the absence of systematic disparities in health (or in the major social determinants of health) between groups with different levels of underlying social advantage/disadvantage – that is, wealth, power, or prestige (21, 22). Knowledge of aggregate effects of multiple vulnerabilities is needed to shed light on the determinants of growing health inequities in Vietnam. This is a repeated cross-sectional study using data from the Vietnam Multiple Indicator Cluster Surveys (MICS) 2000, 2006, and 2011. The aim of this paper is to describe patterns of inequity in maternal healthcare utilization and analyze associations between multiple vulnerabilities and these patterns.

## Methods

### Data source

Data from the MICS 2000, 2006, and 2011 were used for this paper. The MICS were conducted by the General Statistics Office in collaboration with the MOH and the Ministry of Labor, Invalids and Social Affairs. Financial and technical supports for the survey were provided by the United Nations Children's Fund and the United Nations Population Fund. The MICS are nationally representative surveys covering a broad range of issues affecting the health, development, and living conditions of Vietnamese women and children. The number of women who completed interviews in the MICS were 9,117 in 2000; 9,473 in 2006; and 11,614 in 2011 (23–25).

### Variables and indicators

Using available data from all three rounds of the MICS, we derived two binary maternal healthcare utilization indicator variables referring to the receipt of skilled care during the antenatal period and skilled care for birth (delivery). Skilled antenatal care indicates whether a woman had received antenatal care by skilled staff (doctor, nurse, or midwife). Skilled care for deliveries indicates whether an obstetric delivery was assisted by skilled personnel (as defined above).

The explanatory variables are as follows: 1) woman's age in years; 2) woman's education; 3) economic status (measured by household asset-based wealth quintiles); 4) place of residence (rural vs. urban); and 5) woman's ethnicity. Four types of vulnerabilities were analyzed: 1) low education (yes=1 if a woman had primary education or less, no=0 otherwise); 2) poor (yes=1 if a woman belonged to the poorest wealth quintile, no=0 otherwise); 3) rural (yes=1 if a woman lived in a rural area, no=0 otherwise); and 4) ethnic minority (yes=1, no=0 otherwise).

Having no education or only a primary-level education was grouped into the same category, described as ‘low education’, and women with lower secondary, upper secondary, tertiary education were included in another grouping, giving a binary variable for education. The economic condition of households was measured as an asset-based wealth index, constructed using principal component analysis (PCA). Five categories (quintiles) were used – poor, near poor, average, better off, and wealthy – as demonstrated elsewhere (26). We chose women living in rural areas as vulnerable because people living in rural Vietnam normally have lower living standards compared with urban dwellers (30). In Vietnam, ethnic minorities make up 14% of the population and, in general, are relatively disadvantaged in socioeconomic terms (30). The Kinh tribe, which is the major ethnic group in Vietnam, accounts for 86% of the population.

We created two ‘multiple vulnerability’ status indicators: 1) women with three vulnerabilities (yes=1 if a woman had low education *and* belonged to the poorest quintile *and* was living in a rural area; no=0 otherwise) and 2) women with four vulnerabilities (yes=1 if a woman had low education *and* belonged to the poorest quintile *and* was living in a rural area *and* was identified as an ethnic minority; no=0 otherwise).

### Data analysis

Both descriptive and analytical methods were used. The proportions of women who received antenatal care by skilled staff and those who gave birth with assistance from skilled personnel were calculated for all the study respondents, as well as for different groups stratified by vulnerability status. Socioeconomic inequities in maternal healthcare were assessed based on 1) pairwise comparisons and 2) adjusted odds ratios (ORs). In pairwise comparisons, both absolute and relative differences in maternal healthcare indicators between the most advantaged and most disadvantaged population groups were estimated and compared (27). Adjusted ORs were computed by multivariable logistic regression of associations between the maternal healthcare indicators (skilled antenatal and delivery care as dependent variables) and the explanatory variables (women's age and different vulnerability variables). Vulnerabilities were analyzed both as separate explanatory variables and as composite indicators (combinations of the three and four vulnerabilities). The significance level was set at *p*<0.05. We tested for correlation between the individual vulnerability measures and between the combinations and found that they were somewhat correlated, but the correlation coefficients were still lower than 0.7 (a level of strong linear relationship). We also performed univariate analysis and the findings were quite consistent with the multivariable logistic regressions.

## Results

**Fig. 1:**
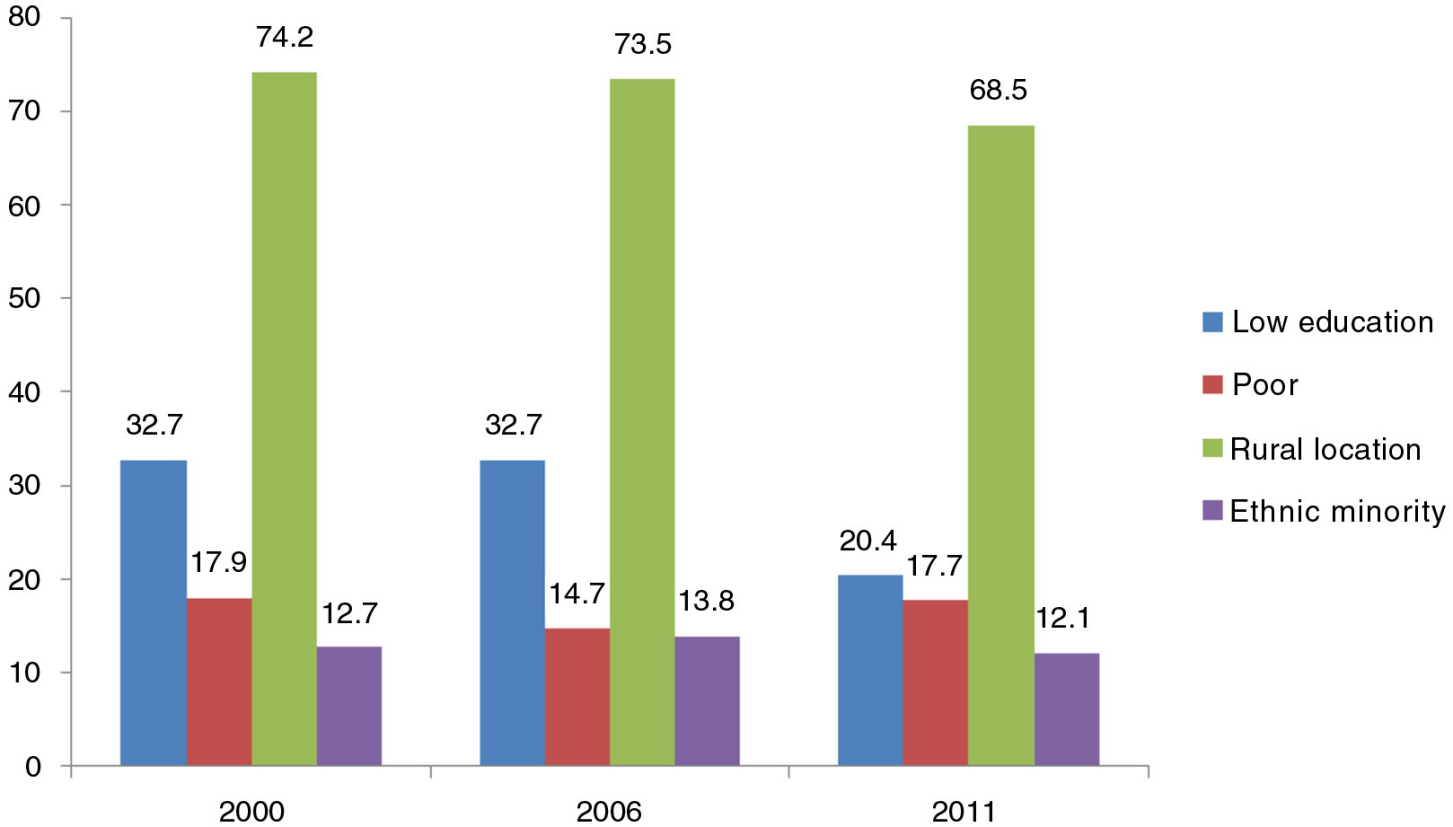
Proportion of women with different types of vulnerabilities in Vietnam (2000, 2006, and 2011).

Figure 1 presents the distribution of the study respondents by type of vulnerability. The proportions of women with low education in 2000, 2006, and 2011 were 32.7, 37.9, and 20.4%, respectively. Ethnic minority women included in the 2000, 2006, and 2011 surveys were 12.7, 13.8, and 12.1%, respectively. Poor women accounted for 17.9, 14.7, and 17.7% of the MICS 2000, 2006, and 2011 samples, respectively. The percentages of women who lived in rural areas in the 2000, 2006, and 2011 surveys were 74.2, 73.5, and 68.5%, respectively.

**Fig. 2:**
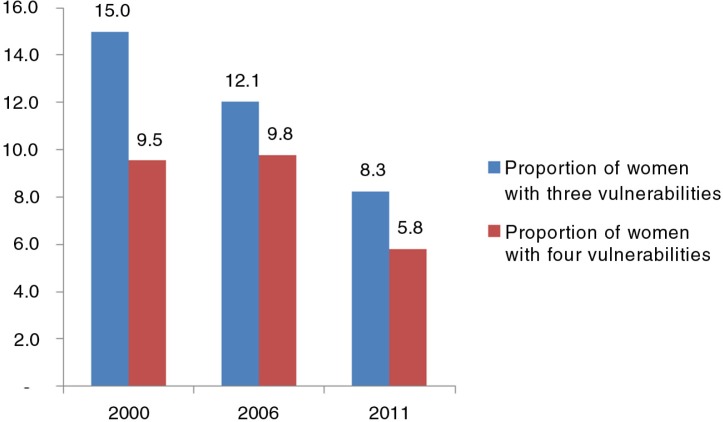
Proportion of women with three and four vulnerabilities in Vietnam (2000, 2006, and 2011). Three vulnerabilities: women having low education *and* belonging to the poorest quintile *and* living in a rural area. Four vulnerabilities: women having low education *and* belonging to the poorest quintile *and* living in rural area *and* identified as ethnic minority.

As shown in Fig. 2, the proportion of women with three specific vulnerabilities (low education level, poorest wealth quintile, and living in a rural area) in 2000, 2006, and 2011 were 15.0, 12.1, and 8.3%, respectively. The percentages of women with four specific vulnerabilities (low education level, poorest wealth quintile, living in a rural area, and identification with an ethnic minority) in 2000, 2006, and 2011 were 9.5, 9.8, and 5.8%, respectively.

**Fig. 3:**
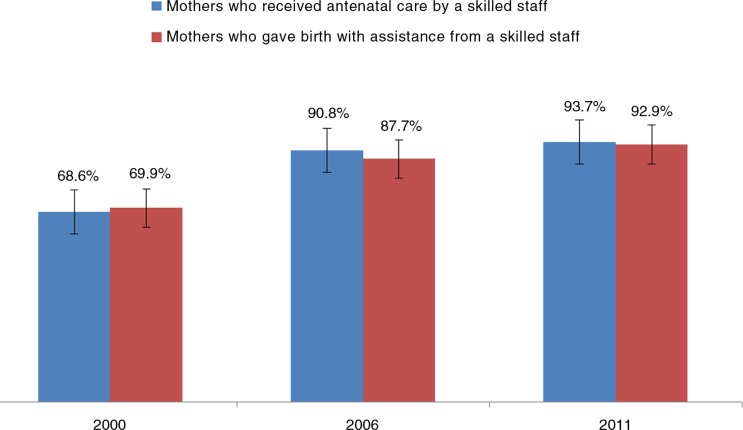
Proportion of women who received antenatal care by skilled staff and those who gave birth with assistance from skilled staff in Vietnam (2000, 2006, and 2011).

Figure 3 shows that, over the three survey years, there were increases in both the percentage of women who received antenatal care by skilled staff (68.6% in 2000, 90.8% in 2006, and 93.7% in 2011) and in the percentage of women who gave birth with assistance from skilled staff (69.9% in 2000, 87.7% in 2006, and 92.9% in 2011).

Table 1 describes the two indicator variables (antenatal care by skilled staff and birth assistance from skilled attendants) by vulnerabilities in 2000, 2006, and 2011. The indicators increased over time. However, in 2011, the proportion of women who received either type of care remained low for those with multiple vulnerabilities. There appeared to be some improvement when comparing individual vulnerabilities, but less so when comparing multiple vulnerabilities (Table 1).

**Table 1:** Proportions of women who received antenatal care by skilled staff and those who gave birth with assistance from skilled attendants by type of vulnerability, Vietnam, 2000, 2006, and 2011

	Antenatal care by skilled staff			Birth with skilled attendant		
		
	2000 (%)	2006 (%)	2011 (%)	2000 (%)	2006 (%)	2011 (%)
Education						
High education	83.3	94.2	96.8	84.1	92.8	97.1
Low education	47.9	87.1	80.8	49.8	82.3	75.3
Economic status						
Non-poor	79.2	96.0	98.0	80.2	95.9	98.7
Poor	40.9	68.5	78.4	43.0	52.8	71.9
Location of residence						
Urban	95.4	98.0	97.9	97.3	98.3	98.8
Rural	62.5	88.6	92.0	63.7	84.5	90.5
Ethnicity						
Kinh	78.2	96.5	97.7	81.3	96.4	98.6
Ethnic minority	26.3	63.2	73.2	19.5	45.8	63.4
Having three vulnerabilities^a^						
Women without any of the three vulnerabilities^a^	78.3	94.6	96.6	79.5	93.6	97.0
Women with all three vulnerabilities^a^	31.6	60.6	66.6	33.4	42.0	53.6
Having four vulnerabilities^b^						
Women without any of the four vulnerabilities^b^	76.0	94.5	96.6	77.9	93.3	96.9
Women with all four vulnerabilities^b^	13.6	52.7	54.3	10.4	30.0	37.7

a Three vulnerabilities: women having low education *and* belonging to the poorest quintile *and* living in a rural area.

b Four vulnerabilities: women having low education *and* belonging to the poorest quintile *and* living in a rural area *and* identified as being an ethnic minority.

Table 2 indicates that the magnitude of inequities, measured by both absolute and relative percentage differences, decreased over time but remained considerable. For example in 2011, comparing the women with all three vulnerabilities (having low education *and* belonging to the poorest quintile *and* living in a rural area) with those without any of the three vulnerabilities, the absolute and relative differences in the percentages receiving antenatal care by skilled staff were 30% and 3.22 times, respectively. The absolute and relative percentage differences for giving birth with assistance from skilled staff were 43.4% and 2.24 times, respectively. Comparing women with all four vulnerabilities (having low education *and* belonging to the poorest quintile *and* living in a rural area *and* identified as an ethnic minority) with those without any of the four vulnerabilities, the absolute and relative percentage differences for antenatal care by skilled staff were 42.3% and 2.28 times, respectively. Absolute and relative percentage differences for giving birth with assistance from skilled staff were 59.2% and 1.64 times, respectively (Table 2).

**Table 2:** Socioeconomic inequalities in skilled antenatal and delivery care, Vietnam, 2000, 2006, and 2011

	Antenatal care by skilled staff			Birth with skilled attendant		
		
	2000	2006	2011	2000	2006	2011
Education						
Absolute difference	35.4%	7.1%	16.0%	34.3%	10.5%	21.8%
Relative difference	2.35	13.27	6.05	2.45	8.84	4.45
Economic status						
Absolute difference	38.3%	27.5%	19.6%	37.2%	43.1%	26.8%
Relative difference	2.07	3.49	5.00	2.16	2.23	3.68
Location of residence						
Absolute difference	32.9%	9.4%	5.9%	33.6%	13.8%	8.3%
Relative difference	2.90	10.43	16.59	2.90	7.12	11.90
Ethnicity						
Absolute difference	51.9%	33.3%	24.5%	61.8%	50.6%	35.2%
Relative difference	1.51	2.90	3.99	1.32	1.91	2.80
Having three vulnerabilities^a^						
Absolute difference	46.7%	34.0%	30.0%	46.1%	51.6%	43.4%
Relative difference	1.68	2.78	3.22	1.72	1.81	2.24
Having four vulnerabilities^b^						
Absolute difference	62.4%	41.8%	42.3%	67.5%	63.3%	59.2%
Relative difference	1.22	2.26	2.28	1.15	1.47	1.64

a Three vulnerabilities: women having low education *and* belonging to the poorest quintile *and* living in a rural area.

b Four vulnerabilities: women having low education *and* belonging to the poorest quintile *and* living in a rural area *and* identified as being an ethnic minority.

Table 3 shows associations between multiple vulnerabilities and the two indicators of maternal healthcare utilization in the three survey years. The odds of receiving skilled antenatal care among women without any of the three vulnerabilities (having low education *and* belonging to the poorest quintile *and* living in a rural area) were much higher than that the odds for women with all three vulnerabilities, and the point estimates of odds ratios increased over time (ORs: 7.86 in 2000, 14.13 in 2011). The odds of receiving skilled care for delivery among women without any of the three vulnerabilities were also higher than those for women having all three vulnerabilities, and the point estimates of odds ratios increased over time (for 2000, 2006, and 2011, ORs were 7.85, 19.47, and 28.11).

**Table 3:** Multivariable logistic regression analysis of relationships between skilled antenatal and delivery care and multiple vulnerabilities, Vietnam, 2000, 2006, and 2011

	Antenatal care by skilled staff			Birth with skilled attendant		
		
	2000 OR (95% CI)	2006 OR (95% CI)	2011 OR (95% CI)	2000 OR (95% CI)	2006 OR (95% CI)	2011 OR (95% CI)
Three vulnerabilities^a^						
Women with all three vulnerabilities^a^	1	1	1	1	1	1
Women without any of the three vulnerabilities^a^	7.86 (4.69–13.17)	11.35 (6.94–18.56)	14.13 (8.49–23.52)	7.85 (4.69–13.14)	19.47 (12.12–31.27)	28.11 (16.62–47.54)
Four vulnerabilities^b^						
Women with all four vulnerabilities^b^	1	1	1	1	1	1
Women without any of the four vulnerabilities^b^	19.98 (9.43–42.35)	15.13 (9.07–25.22)	23.34 (13.6–40.04)	29.92 (12.01–74.52)	31.6 (18.84–53.01)	50.32 (28.23–89.70)

*Notes:* ORs were adjusted for the age of the women; OR, odds ratio; CI, confidence interval.

a Three vulnerabilities: women having low education *and* belonging to the poorest quintile *and* living in a rural area.

b Four vulnerabilities: women having low education *and* belonging to the poorest quintile *and* living in a rural area *and* identified as ethnic minority.

Table 3 shows that magnitudes of inequities in the two indicators were much higher when comparing women with the four selected vulnerabilities (having low education *and* belonging to the poorest quintile *and* living in a rural area *and* identified as an ethnic minority) with women without any of the four vulnerabilities. The odds of receiving skilled antenatal care among women without any of the four vulnerabilities in 2000, 2006, and 2011 were 19.98, 15.13, and 23.34 times higher than the odds for women with all four vulnerabilities. The odds of receiving skilled care during deliveries for women without any of the four vulnerabilities in 2000, 2006, and 2011 were 29.92, 31.6, and 50.32 times higher than the reference group – women with all four vulnerabilities (Table 3).

## Discussion

This study provides additional research evidence about patterns of inequity in two indicators of maternal healthcare utilization specified in MDG5 (improve maternal health). The antenatal period is very important for the health of the mother and her baby. Antenatal visits with skilled health staff can help prevent, diagnose, and treat many potential problems arising during pregnancy. Skilled attendance during delivery is also critical for improved maternal outcomes. Many obstetric delivery complications can be prevented or better managed if deliveries are assisted by skilled attendants (25).

Our analyses show that although there have been improvements in the coverage of skilled antenatal and delivery care in Vietnam, these improvements have been uneven. Considerable socioeconomic inequities in maternal healthcare utilization in both the antenatal and delivery phases still exist. This finding is in line with previous research in Vietnam, which showed that low educational levels, poverty, and ethnicity were significantly associated with lower antenatal care coverage and the attendance of skilled staff during deliveries (11–13, 28).

Of particular interest in this paper is the association between multiple vulnerabilities and inequities in maternal healthcare utilization in Vietnam. The concept of multiple vulnerabilities is quite similar to the ‘multi-dimensional poverty’ notion that has been developed and discussed extensively in Vietnam. The multidimensional vulnerabilities approach can serve to identify targets for poverty reduction programs, as well as tools to monitor the status of poverty reduction both locally and nationally (17, 29). We were interested in analyzing four specific vulnerabilities (low education, poverty, living in a rural area, and ethnic minority identity), because they are among the key priority areas identified by poverty reduction programs and equity-oriented interventions in Vietnam (17). Although research has shown that each of these vulnerabilities is independently associated with inequity in maternal healthcare in Vietnam (11–13, 28), there has to date been no analysis of the impact of combinations of these vulnerabilities on maternal healthcare utilization in Vietnam.

We found that women with multiple vulnerabilities (low education, ethnic minority identity, poverty, and living in a rural area) had much lower likelihoods of receiving antenatal care by skilled staff and giving birth with the assistance of skilled personnel. Similar findings have also been reported in some international research studies. A 2005 study conducted in the United States, for example, showed that multiple vulnerabilities (including low income, no health insurance, and lack of regular source of care) were significantly associated with higher risk of having unmet healthcare needs (30). Another US study, conducted in 2006, showed that the accumulation of social disadvantage (including poverty, minority race/ethnicity, low parental education, and not living with both biological parents) among children was strongly related to poorer child health (31). A 2008 study in Ghana, Kenya, and Ethiopia investigated the compound effect of dual forms of vulnerability (poverty status, education, region, and ethnicity) and showed that multiple forms of marginalization often confer greater risk. These results suggest that in order to fine-tune policies to reach the most marginalized, simple allocation formulas targeting a particular region or ‘the poor’ are not sufficiently nuanced (29). A study conducted in India in 2012 reported that women with multiple vulnerabilities were less likely to receive skilled antenatal care and delivery with medical assistance (16).

We need to note this study's limitations. The two key indicators within MDG5 are ANC1 (at least one visit with a skilled provider) and ANC4 (at least four visits with any provider). However, due to the available MICS data and in order to ensure comparability across survey years, this study focused only on ANC1. Another limitation is the wide confidence intervals in the estimates, making it difficult to accurately determine secular trends in maternal healthcare inequities and multiple vulnerabilities. In addition, due to the paucity of research in this area, it was difficult to relate these findings to a wide body of literature, but we have nonetheless referred to known similar studies. There is clearly a need for further research in this area. The study design was also a limitation. A longitudinal design would have allowed a follow-up of each individual with multiple vulnerabilities. For example, we were unable to say whether there were new individuals with these measured vulnerabilities, if some women dropped out of the surveys, or the extent to which women with multiple vulnerabilities remained in the surveys.

However, this study also has some strengths. Importantly we identified multiple socioeconomic vulnerabilities related to maternal healthcare utilization using nationally representative data. We have brought to light information from the MICS that was not otherwise apparent from the survey results. This study showcases the use of indicators that depict multiple socioeconomic vulnerabilities in relation to maternal healthcare in a developing country.

## Conclusions

In conclusion, this study shows that there have been significant improvements in both skilled antenatal care and skilled care during deliveries in Vietnam over recent years. However inequities in these two important maternal healthcare utilization indicators persist. Women with multiple vulnerabilities were less likely to have access to essential maternal healthcare. Actions to address inequities in skilled antenatal and delivery care, as well as access to general healthcare, should be comprehensive and based on multi-sectoral approaches. The evidence from this research can inform policy makers in Vietnam in designing and implementing interventions to improve maternal healthcare in Vietnam and to achieve MDG5 in a more efficient and equitable manner. We suggest that researchers undertaking studies on the social determinants of health should consider the use of composite indices of multiple socioeconomic vulnerabilities, such as demonstrated here.
